# Cold exposure induces lipid dynamics and thermogenesis in brown adipose tissue of goats

**DOI:** 10.1186/s12864-022-08765-5

**Published:** 2022-07-21

**Authors:** Xin Liu, Jing Tang, Runan Zhang, Siyuan Zhan, Tao Zhong, Jiazhong Guo, Yan Wang, Jiaxue Cao, Li Li, Hongping Zhang, Linjie Wang

**Affiliations:** 1 Farm Animal Genetic Resources Exploration and Innovation Key Laboratory of Sichuan Province, Sichuan Agricultural University, Chengdu, 611130 Sichuan People’s Republic of China; 2 College of Animal Science and Technology, Sichuan Agricultural University, Chengdu, 611130 Sichuan People’s Republic of China

**Keywords:** Brown adipose tissue, Cold exposure, Lipid metabolism, RNA-seq, Thermogenesis

## Abstract

**Background:**

Adaptive thermogenesis by brown adipose tissue (BAT) is important to the maintenance of temperature in newborn mammals. Cold exposure activates gene expression and lipid metabolism to provide energy for BAT thermogenesis. However, knowledge of BAT metabolism in large animals after cold exposure is still limited.

**Results:**

In this study, we found that cold exposure induced expression of BAT thermogenesis genes and increased the protein levels of UCP1 and PGC1α. Pathway analysis showed that cold exposure activated BAT metabolism, which involved in cGMP-PKG, TCA cycle, fatty acid elongation, and degradation pathways. These were accompanied by decreased triglyceride (TG) content and increased phosphatidylcholine (PC) and phosphatidylethanolamine (PE) content in BAT.

**Conclusion:**

These results demonstrate that cold exposure induces metabolites involved in glycerolipids and glycerophospholipids metabolism in BAT. The present study provides evidence for lipid composition associated with adaptive thermogenesis in goat BAT and metabolism pathways regulated by cold exposure.

**Supplementary Information:**

The online version contains supplementary material available at 10.1186/s12864-022-08765-5.

## Introduction

Tremendous progress has been made in the study of adipose tissue thermogenesis. Unlike shivering thermogenesis of muscles, brown adipose tissue (BAT) can generate large amounts of heat through non-shivering thermogenesis, which is mainly performed by UCP1 uncoupled respiration [1]. In rodents, BAT is formed during embryonic development and persists in adulthood to maintain body temperature [2, 3]. Interscapular BAT is the largest brown fat depot of adult mice and contributes to its adaptive thermogenesis [4]. However, the developmental regulation of brown fat in rodents is different from that in large mammals. In sheep and goats, BAT is present in clavicular/cervical, pericardial, perirenal regions and the largest brown fat depot was the perirenal fat at birth. Then, it is converted from BAT to white adipose tissue (WAT) at 30 days after birth [5–7]. Perirenal BAT is recruited only at birth to help newborn mammals adapt to changes in ambient temperature [8].

When exposed to cold, BAT is recruited for adaptive thermogenesis, which is important for newborn mammals to maintain body temperature in the extrauterine environment [9]. Additionally, genes involved in lipolysis are upregulated upon clod exposure, where cold activates HSL and ATGL via β-adrenergic receptors to supply huge energy for brown fat thermogenesis [10]. Meanwhile, Lipid metabolism is essential for BAT thermogenesis in response to cold exposure [11]. BAT generates energy as heat and mobilizes fatty acids from the TG in lipid droplets to mitochondria for thermogenesis to increase body temperature [12]. Previous studies have been proved that cold exposure causes huge changes in the expression of genes involved in glycerophospholipid metabolism of interscapular BAT of mice [13]. There are huge changes in the species composition of glycerophospholipids as well as TG. Cold induced fatty acyl chain elongation of glycerolipids, while glycerophospholipid species are generally upregulated [13]. Meanwhile, phosphatidylglycerol (PG) is upregulated in cold conditions and may serve as a marker of BAT activity [14]. However, as the largest brown fat depot in the postnatal period, cold-induced lipid metabolism in perirenal BAT remains largely unknown in large mammals.

In this study, we performed acute cold exposure (24 h at 6 °C) on newborn goats by contrasting the room temperature group (24 h at 25 °C). We found that cold exposure altered the transcription pattern of perirenal BAT from newborn goats. The genes involved in cGMP-PKG signaling, TCA cycle, and PPAR signaling pathway were widely upregulated, suggesting that those pathways were activated after cold exposure. Furthermore, lipidomic analysis of perirenal BAT indicated that cold elicited pathways of glycerolipid and glycerophospholipid metabolism. This study provides comprehensive data on gene expression and lipid composition of goat perirenal BAT after cold exposure. These datasets will be useful for further research on BAT lipid metabolism in large mammals after cold exposure.

## Result

### Cold exposure promotes BAT thermogenesis and induces the transcriptional programs of perirenal BAT in newborn goats

**Fig. 1:**
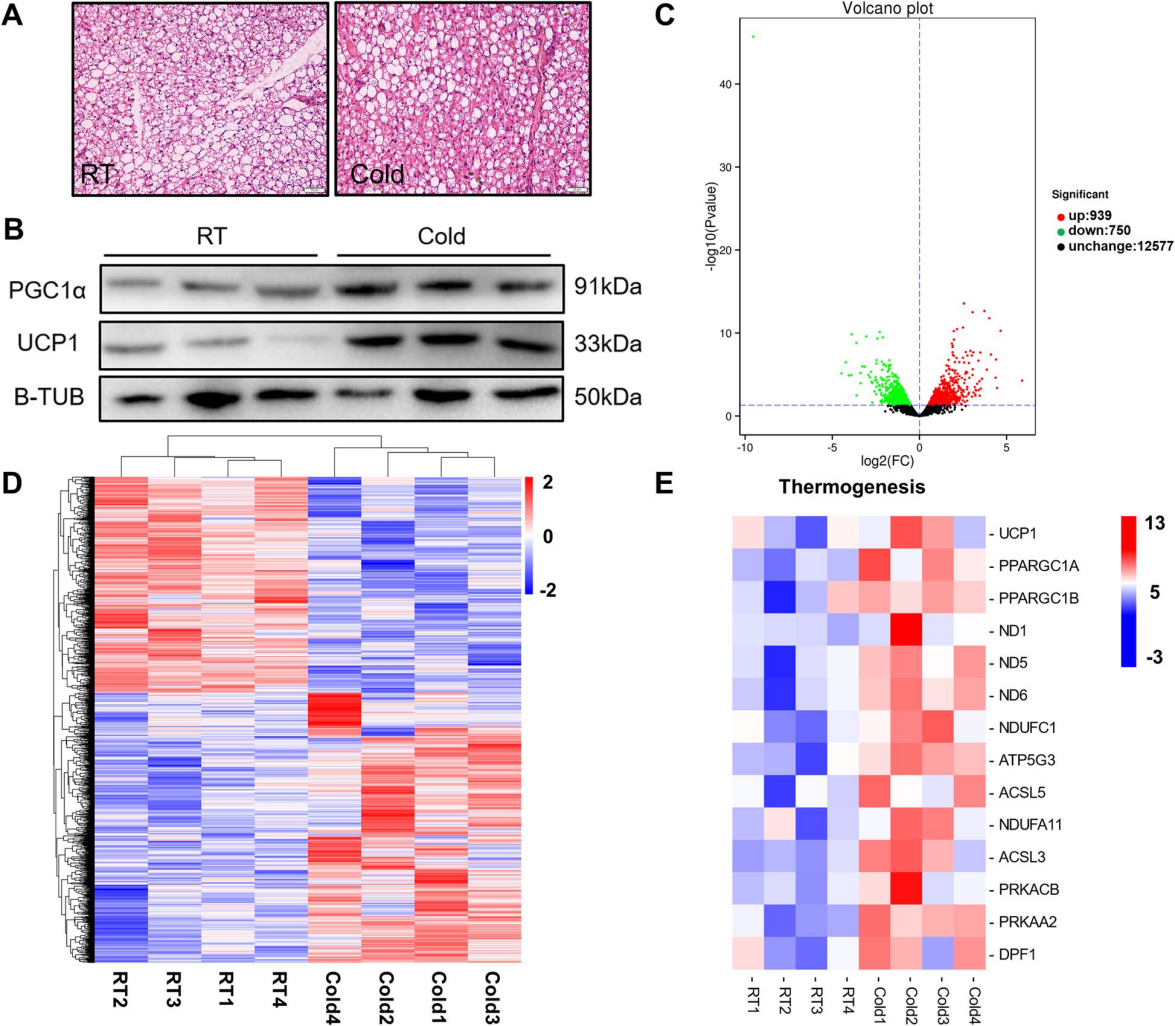
Cold exposure promotes BAT thermogenesis of perirenal BAT in newborn goats. (**A**) Representative images are shown for perirenal fat and histological sections were stained with hematoxylin and eosin of room temperature (RT, 25 °C) or cold exposure (Cold, 6 °C) group, scale bar: 50 μm; (**B**) Western blotting of UCP1 and PGC1α between RT and Cold group in BAT; (**C**) Volcano plot showed differential gene expression profiles of RT and Cold group; (**D**) Unsupervised hierarchical clustering showed that the RT and Cold groups clustered into two classes; (**E**) Heatmaps of the fragments per kilobase million (FPKM) values of upregulated genes in BAT thermogenesis pathway after cold exposure

In this study, we first carried out HE staining to characterize the tissue morphology of perirenal BAT. The individual cell compartment of perirenal BAT was reduced after cold exposure (Fig. 1A). Besides, cold exposure significantly increased the protein levels of UCP1 and PGC1α (Fig. 1B and Fig. S1). Next, we determined changes of gene transcriptomic profile induced by cold exposure in perirenal BAT through RNA-seq (Table S1). A total of 1,689 differentially expressed genes were obtained, of which 939 and 750 genes were upregulated and downregulated by cold exposure (Fig. 1C and Table S2). Unsupervised hierarchical clustering of differentially expressed genes generated two main clusters between room temperature (RT) and cold exposure (Cold) group (Fig. 1D), indicate that the samples had good uniformity among the replicates. In addition, we observed that the BAT thermogenesis related genes were significantly upregulated after cold exposure, including *UCP1*, *PGC1α*, *PGC1β*, *ND1*, *ATP5G3*, *ACSL5*, and *DPF1* (*P* < 0.05, Fig. 1E).

**Fig. 2:**
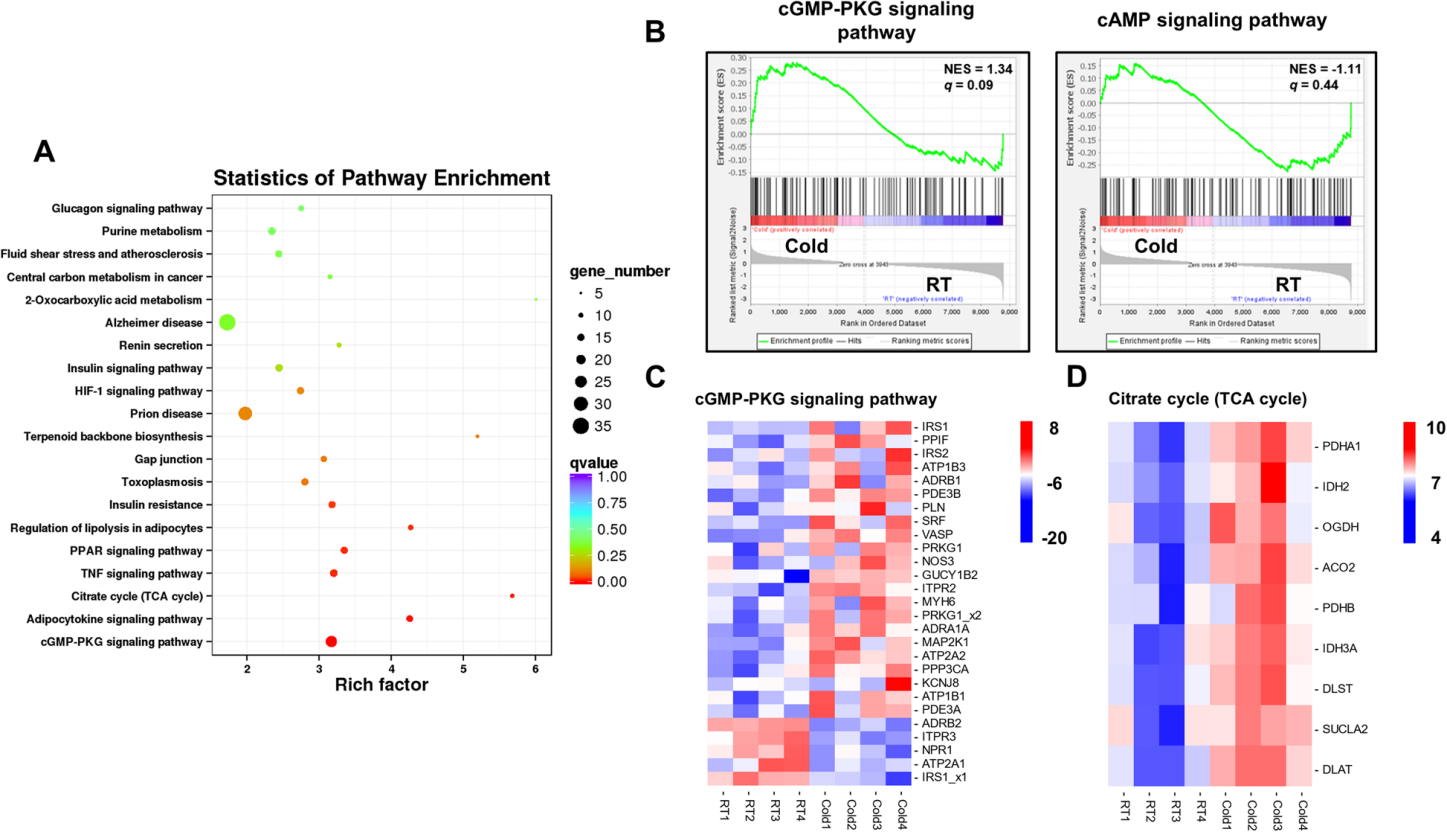
KEGG pathway analysis of upregulated genes of BAT upon cold exposure. (**A**) Enrichment of Kyoto Encyclopedia of Genes and Genomes (KEGG) pathways of cold-induced upregulated genes using KEGG database [16]; (**B**) cGMP-PKG (ko04022) and cAMP signaling pathway (ko04024) analysis was used by gene set enrichment analysis (GSEA) on RNA-seq data from RT and Cold group. The green line represented the enrichment of pathway in the Cold or RT group according to gene expression levels, with genes enriched in the Cold group shown on the left and genes enriched in the RT group shown on the right; (**C**-**D**) Heatmaps of the FPKM expression values of differentially expressed genes in cGMP-PKG signaling pathway and Citrate cycle (TCA cycle) after cold exposure. IRS1_x1 represented IRS1 isoform 1, and PRKG1_x2 represented PRKG1 isoform 2

KEGG pathway analysis revealed that the upregulated genes after cold exposure were enriched in BAT metabolism and thermogenesis-related pathways, including cGMP-PKG signaling, TCA cycle, PPAR, and regulation of lipolysis in adipocytes (Fig. 2A and Table S3). We next characterized these pathways related differentially expressed genes using heatmaps. Gene set enrichment analysis (GSEA) of RNA-seq data showed that the cGMP-PKG signaling pathway was highly enriched in perirenal BAT after cold exposure, but absent in the cAMP signaling pathway (Fig. 2B). These genes involved in cGMP signaling were significantly upregulated (*P* < 0.05, Fig. 2C), such as *ADRA1A*, *ADRB1*, *NOS3*, *GUCY1B2*, *PRKG1*, *PDE3B*, and *VASP* genes. Norepinephrine (NE) is known to activate β adrenergic receptors (*ADRA1A* and *ADRB1*), which then activate endothelial nitric oxide synthase (*NOS3*). The endothelial nitric oxide synthase catalyzes the production of nitric oxide, which further potentiates activation of soluble guanylate cyclase (*GUCY1B2*). Then GUCY1B2 catalyzes the synthesis of cGMP, and PKG (*PRKG1*) plays a regulatory role through binding cGMP [15]. These results suggest that cGMP-PKG signaling pathway, but not cAMP signaling pathway, was activated by NE-β adrenergic receptors signaling in perirenal BAT upon cold exposure. Furthermore, cold exposure induced the expression levels of the genes encoding the rate-limiting enzymes for TCA cycle, including aconitate hydratase (ACO2), isocitrate dehydrogenase (*IDH2*), pyruvate dehydrogenase complex (*PDHA1* and *PDHB*), and the ketoglutarate dehydrogenase complex (*OGDH* and *DLST*) (Fig. 2D). The above finding mirrored at the transcriptional level that cGMP-PKG signaling pathway and TCA cycle was elicited in perirenal BAT upon cold exposure.

### Cold exposure changes the overall lipid composition of perirenal BAT

**Fig. 3:**
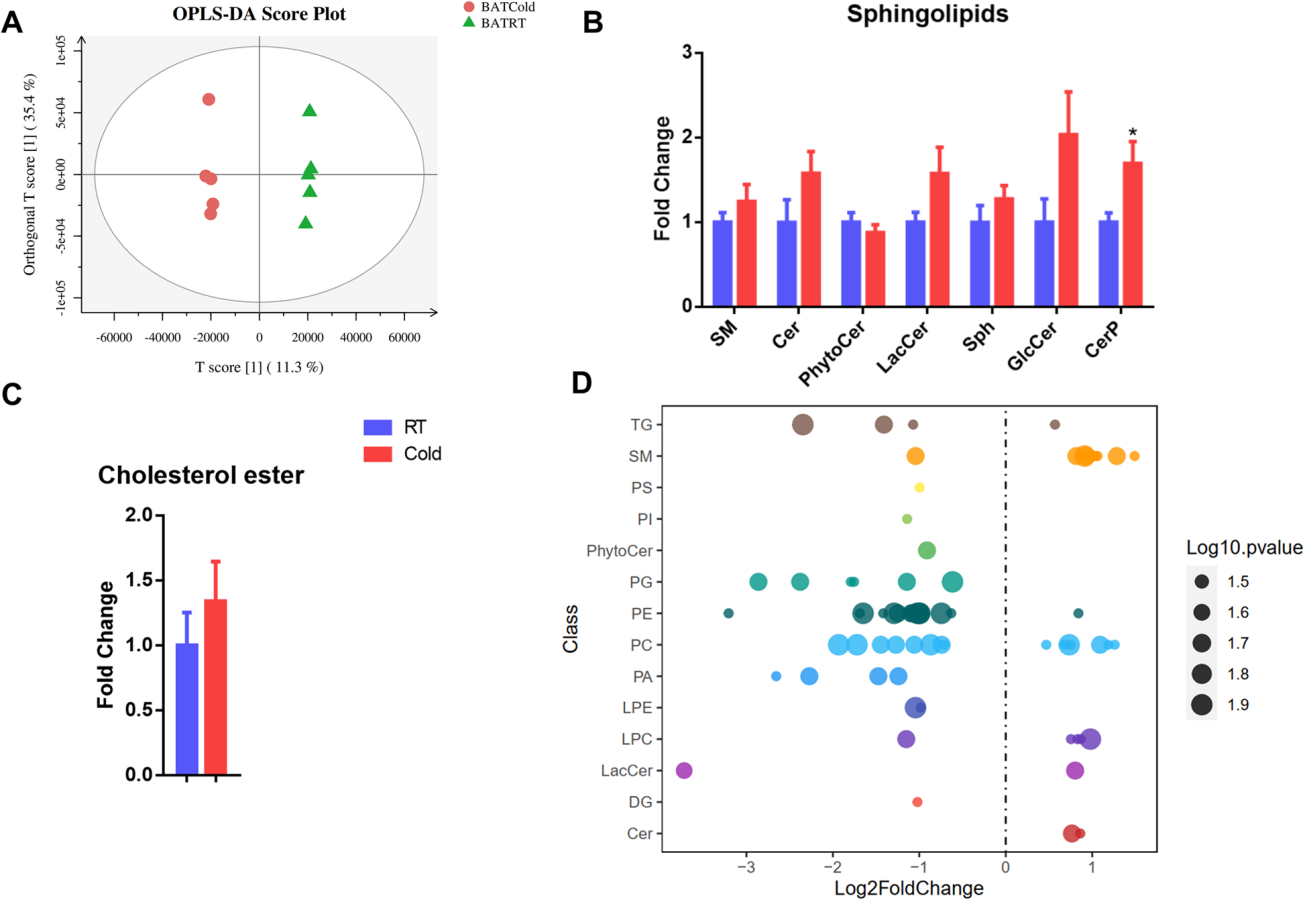
Cold exposure changes the overall lipid composition of perirenal BAT. (**A**) The orthogonal partial least squares-discriminant analysis (OPLS-DA) showed that RT and Cold group were separated into two clusters; (**B**-**C**) The intensity fold change of sphingolipids and cholesterol esters; (**D**) Log2 fold changes in lipid species in Cold vs. RT group. Each dot represents a lipid species and the dot size indicates the significance. Only lipids with *P* < 0.05 are displayed. Error bars represent standard error of mean (SEM), *n* = 5, * *P* < 0.05

LS-MS-based lipidomics was used to quantify the levels of lipid in perirenal BAT from cold exposure and room temperature group. A totally of 1469 different lipid species were detected in BAT (Table S4). There was an obvious separation of cold exposure and room temperature group by the orthogonal projections to latent structures discriminant analysis (Fig. 3A). For sphingolipids, only ceramides phosphate (CerP) was significantly increased (*P* < 0.05, Fig. 3B). Cholesterol esters (CE) were unchanged under cold exposure (Fig. 3C). Then, we used bubble plots to visualize all significantly different lipid species. A total of 68 lipid species were significantly changed, of which 25 and 43 lipid species were upregulated and downregulated by cold exposure, respectively (*P* < 0.05, Fig. 3D and Table S5).

### Cold exposure changes fatty acyl levels of TG in perirenal BAT

**Fig. 4:**
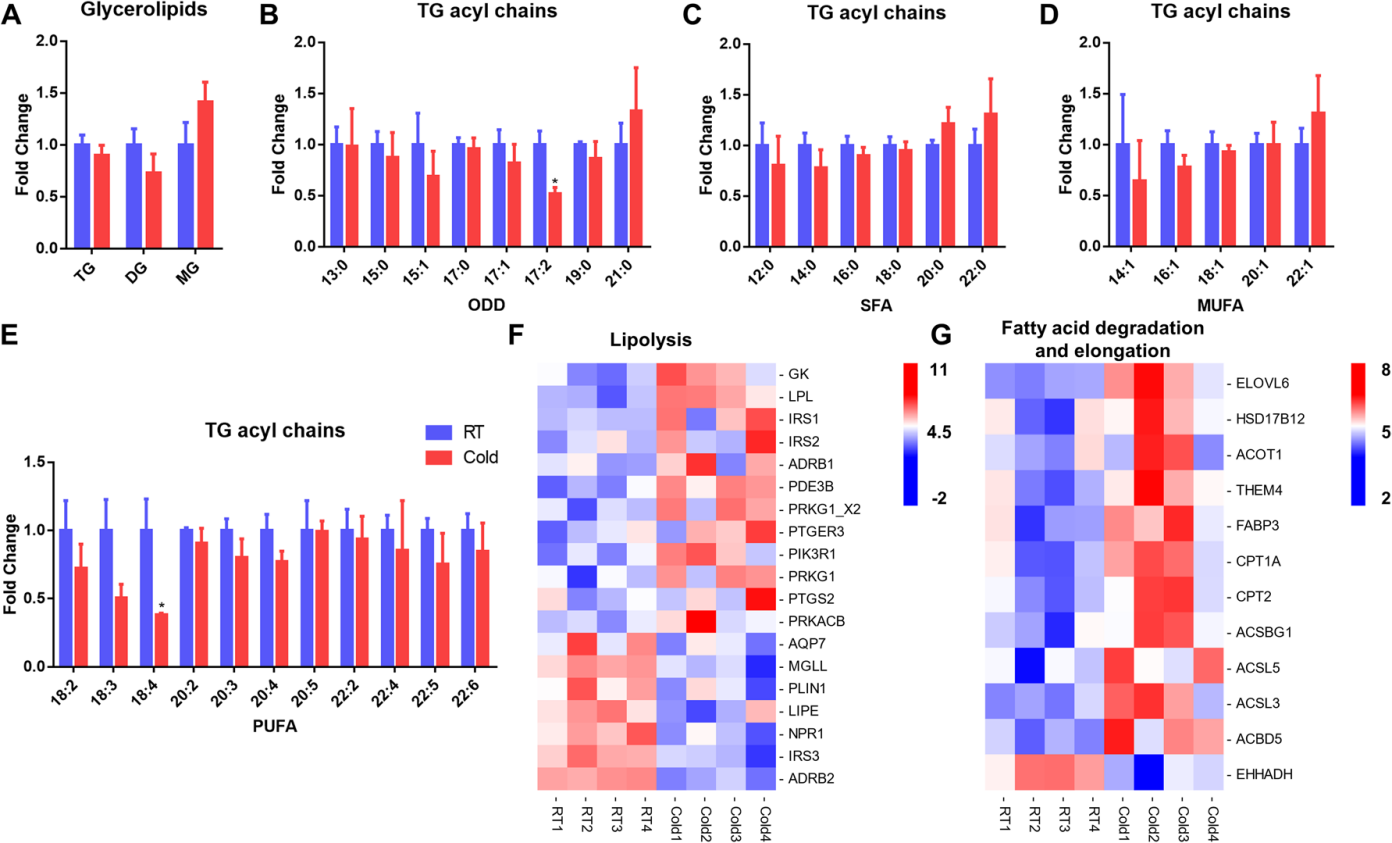
Cold exposure changes fatty acyl levels of TG in perirenal BAT. (**A**) The intensity fold change of glycerolipids; (B-E) The total intensity fold change of TG individual fatty-acyls chains, (**B**), odd-numbered fatty-acyl chains (ODD), (**C**), saturated fatty-acyl chains (SFA), (**D**), monounsaturated fatty-acyl chains (MUFA), (**E**), polyunsaturated fatty-acyl chains (PUFA); (**F**-**G**) Heatmaps of the FPKM values of differentially expressed genes in regulation of lipolysis, and fatty acid degradation and elongation pathway after cold exposure. Error bars represent standard error of mean (SEM), *n* = 5, * *P* < 0.05

Our lipidomic results show that TG and diglyceride (DG) were not significantly changed by cold exposure in perirenal BAT, but there was a tendency of lower TG and DG contents in the Cold group (Fig. 4A). We next investigated the composition of individual fatty acyl chain after cold exposure (Table S6). For odd-numbered fatty-acyl chains (ODD), C17:2 significantly reduced by cold exposure (*P* < 0.05, Fig. 4B). Analysis of saturated fatty-acyl chains (SFA) and monounsaturated fatty-acyl chains (MUFA) exhibited an increasing trend of C20:0, C22:0, C22:1 (Fig. 4C and Fig. 4D). The levels of polyunsaturated fatty-acyl chains (PUFA) were reduced, with a significantly decreased of C18:4 (*P* < 0.05, Fig. 4E).

**Fig. 5:**
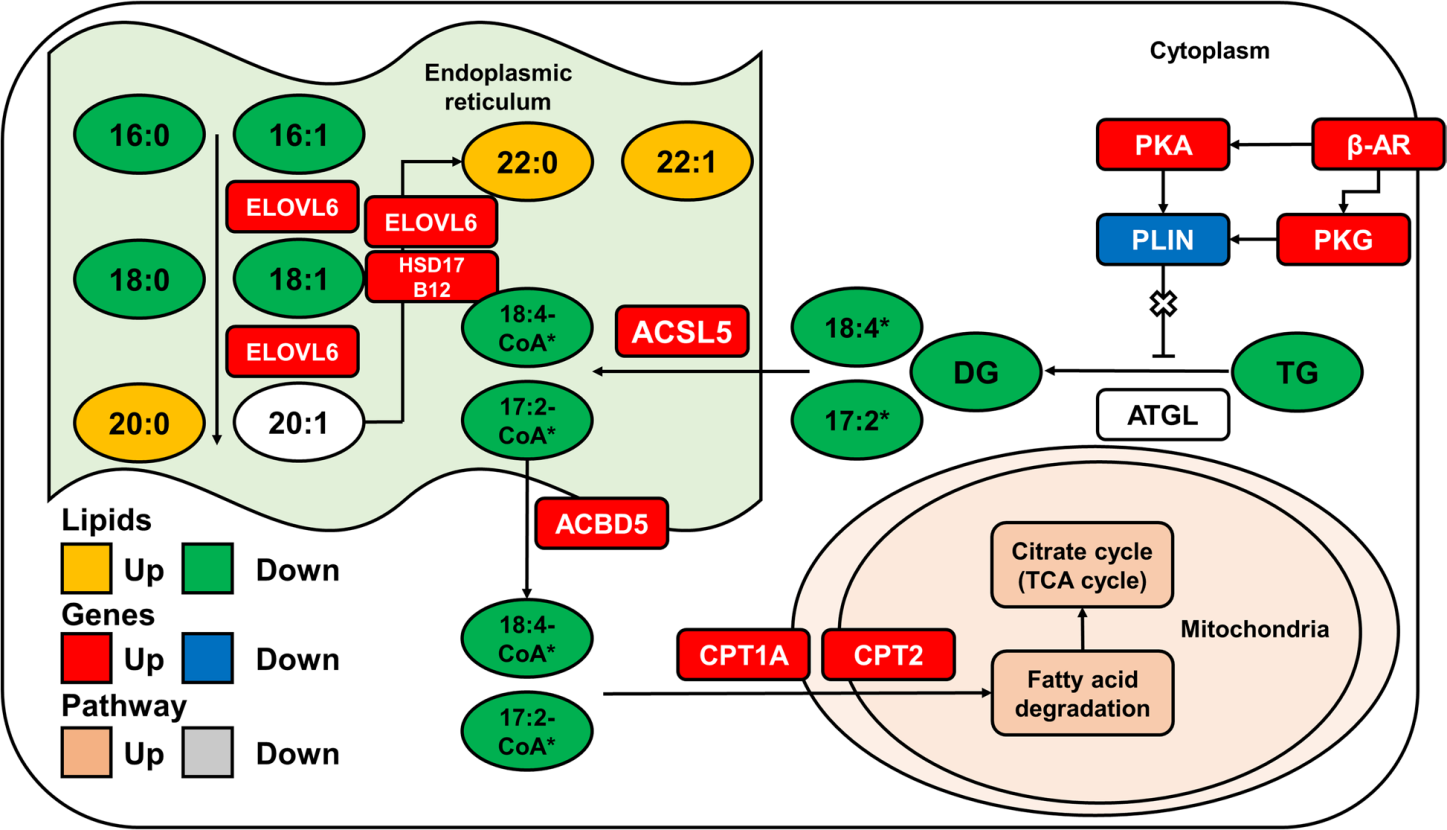
The lipolysis and fatty acid metabolism are induced in perirenal BAT after cold exposure. Pathway analysis of TG lipolysis and fatty acid metabolism, with indications of quantified lipid classes (circles), genes, and pathway (rectangles) regulated in perirenal BAT by acute cold exposure. Colors indicate significantly upregulated (red) or downregulated (blue) genes after cold exposure. For lipids, colors indicate the increasing (yellow) and decreasing (green) trend of the lipid classes, and only * represent significant different lipid classes

Then, we used a heatmap to visualize the genes with significant changes in the lipolysis, fatty acid degradation and elongation pathway (Fig. 4F and Fig. 4G). The expression of *ADRB1*, encoding the β_1_ adrenergic receptor, was significantly upregulated by cold exposure (Fig. 4F, *P* < 0.05). We noticed that expression levels of genes encoding both PKA (*PRKACB*) and PKG (*PRKG1*) were upregulated (Fig. 4F, *P* < 0.05). The expression of *PLIN1* significantly decreased by cold exposure, which functions to protect TG from lipase degradation (Fig. 4F, *P* < 0.05). We found that the expression of *ELOVL6* and *HSD17B12*, which were involved in fatty acid chain elongation, were significantly upregulated by clod exposure (*P* < 0.05, Fig. 4G). Additionally, the *CPT1A* and *CPT2* genes, which are involved in the transport of fatty acids to β-oxidation [17], were significantly upregulated after clod exposure (*P* < 0.05, Fig. 4G). The ACSL5 gene encodes long-chain acyl CoA synthetase, which is a key enzyme for β-oxidation [18]. In this study, ACSL5 was upregulated in BAT after cold exposure (*P* < 0.05, Fig. 4G). These results indicated that fatty acid degradation and fatty acid elongation pathways were activated in perirenal BAT after cold exposure (Fig. 5).

### Cold exposure changes glycerophospholipid metabolism in perirenal BAT

**Fig. 6:**
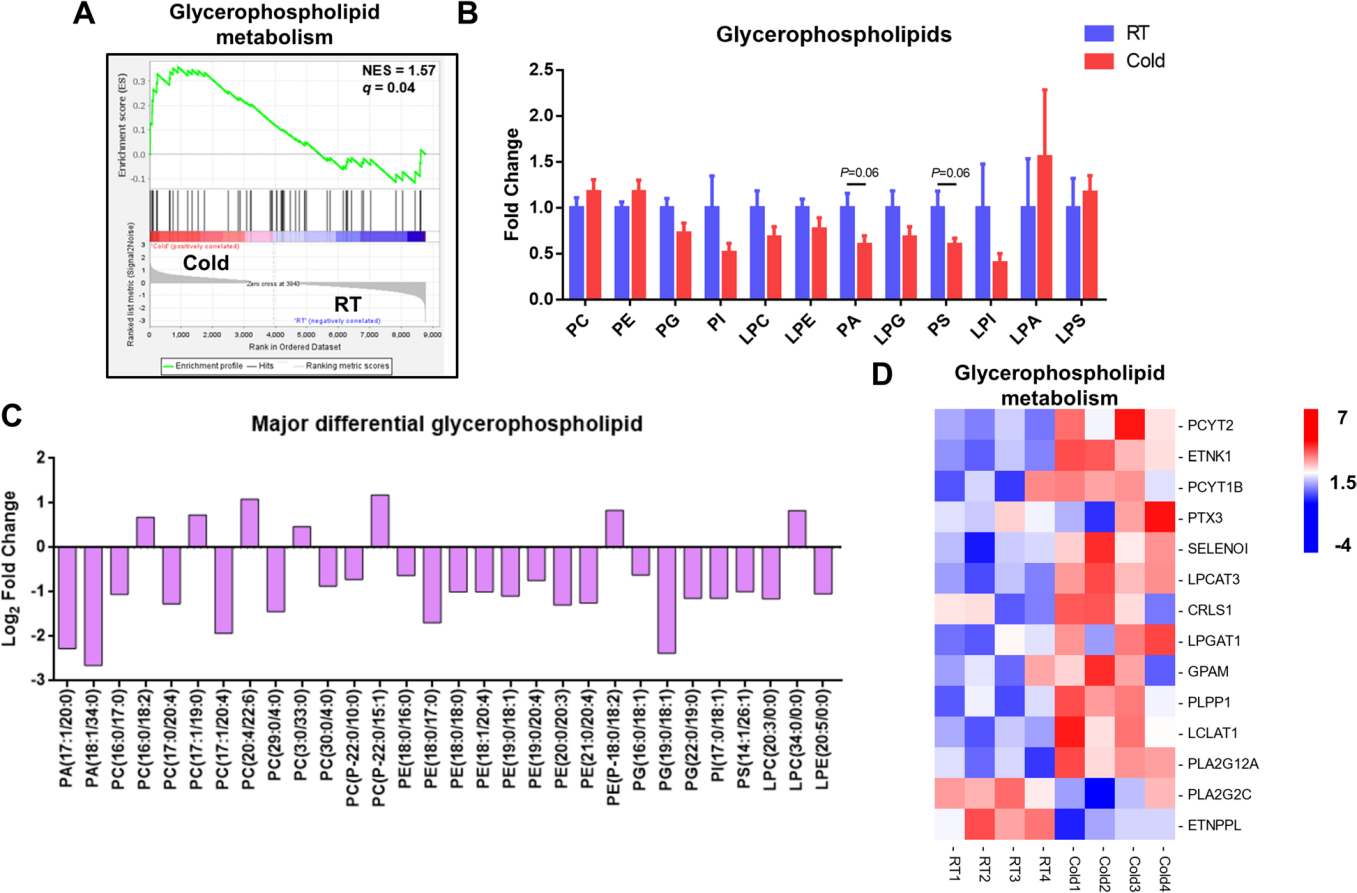
Cold exposure changes glycerophospholipid metabolism in perirenal BAT. (**A**) Glycerophospholipid metabolism (ko00564) pathway analysis using GSEA on RNA-seq data from RT and Cold group. The green line represented the enrichment of pathway in the Cold or RT group according to gene expression levels, with genes enriched in the cold group shown on the left and genes enriched in the RT group shown on the right; (**B**) The intensity fold change of glycerophospholipid; (**C**) All phospholipid species were significantly changed as shown in this figure. Log2 fold changes in significantly different glycerophospholipid species in RT vs. Cold group, and top 30 species are listed according to intensity; (**D**) Heatmaps of the FPKM expression values of upregulated genes in glycerophospholipid metabolism pathway after cold exposure. Error bars represent standard error of mean (SEM), *n* = 5, * *P* < 0.05

GSEA analysis revealed that the upregulated genes were enriched in the glycerophospholipid metabolism pathway after cold exposure (*P* < 0.05, Fig. 6A). As shown in Fig. 6B, phosphatidic acid (PA) and phosphatidylserine (PS) have a decreasing trend, although the difference was not significant (*P* = 0.06). The two most abundant glycerophospholipids (PC and PE) had an upward trend. We next investigated how the composition of the different glycerophospholipid species was affected by cold exposure in perirenal BAT and examined the top 30 differential species according to the intensity (*P* < 0.05, Fig. 6C). The result showed that the content of PE species with C18:0 was significantly decreased (*P* < 0.05), suggesting that these PE species were selected for degradation.

**Fig. 7:**
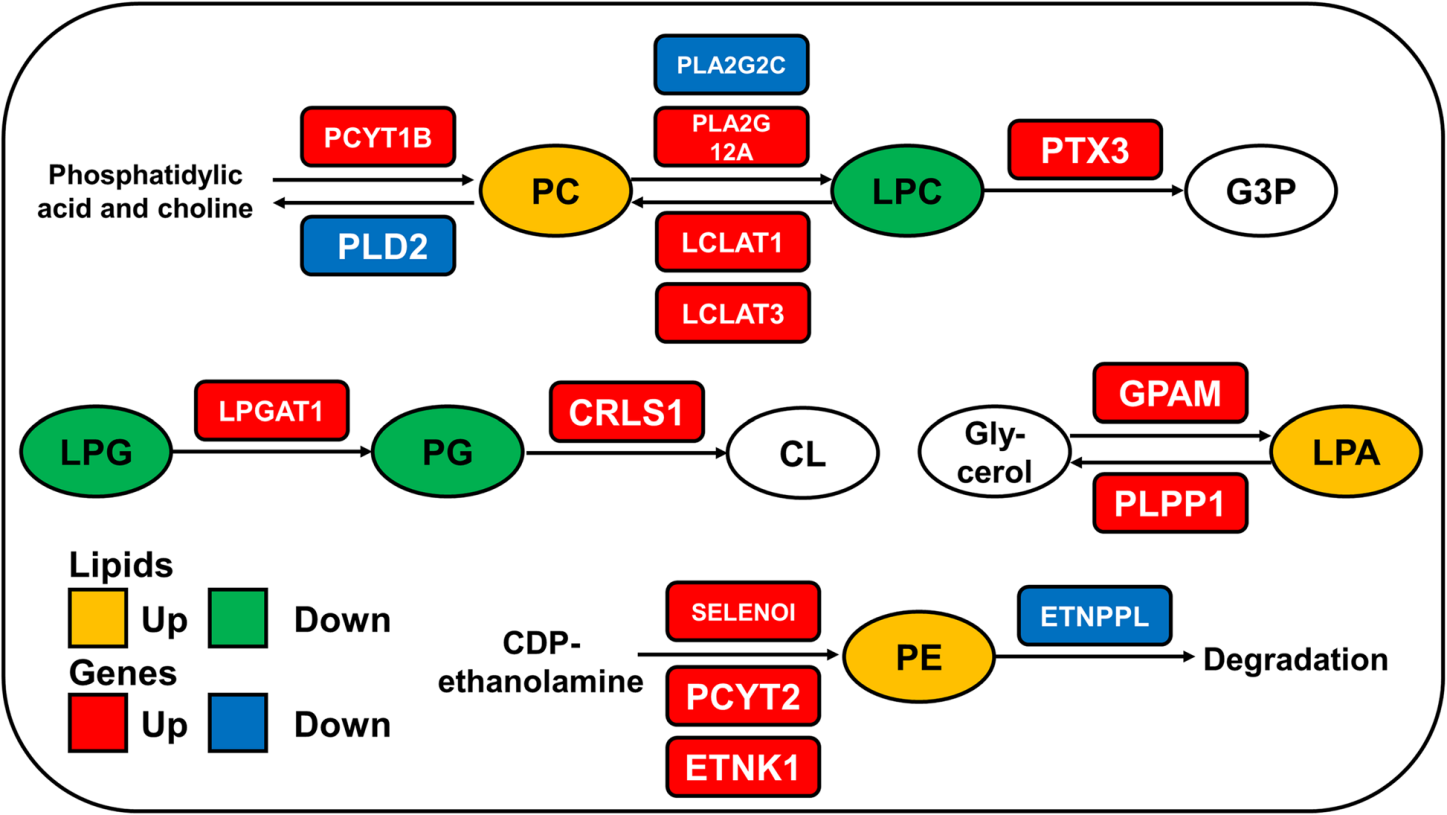
The glycerophospholipid metabolism is induced in perirenal BAT after cold exposure. Pathway analysis of glycerophospholipid metabolism, with indications of quantified lipid classes (circles), genes (rectangles) regulated in perirenal BAT by cold exposure. Colors indicate significantly upregulated (red) or downregulated (blue) genes after cold exposure. For lipids, colors indicate the increasing (yellow) and decreasing (green) trend of the lipid classes. LPA: Lysophosphatidic acid; LPC: Lysophosphatidylcholine; PC: Phosphatidylcholine; PE: Phosphatidylethanolamine; LPG: Lysophosphatidylglycerol; PG: Phosphatidylglycerol; CL: cardiolipin; G3P: Glycerol-3-phosphate

We then used heatmap to show the significantly differentially expressed genes in the glycerophospholipid metabolic pathway from the KEGG analysis (Fig. 6D) and further mapped the pathway between genes and glycerophospholipid species (Fig. 7). The results showed that differentially expressed genes were changed in glycerophospholipid metabolism, which included genes of phosphatidylcholine metabolism-related pathway (*PCYT1B*, *PLA2G12A*, *LCLAT1*, *LCLAT3*, and *PTX3*), cardiolipin synthesis pathway (*LPGAT1* and *CRLS1*), and phosphatidylethanolamine metabolism-related pathway (*SELENOI*, *PCYT2,* and *ETNK1*) (*P* < 0.05, Fig. 6D and Fig. 7). We found that the expression of genes in the PC synthesis was significantly upregulated. Meanwhile, the expression of *PLD2* gene, which encodes the key enzyme for PC degradation, was significantly decreased (*P* < 0.05). In addition, the expression of genes (*SELENOI*, *PCYT2*, and *ETNK1*), which were involved in the PE synthesis, were upregulated, whereas genes involved in the PE degradation were downregulated (*P* < 0.05, Fig. 6D and Fig. 7). These results explain the increased content of PC and PE in BAT after cold exposure.

## Discussion

Previous studies have demonstrated that 22 °C is used as thermoneutral condition of control group for mice cold exposure experiments [13, 19, 20]. In addition, 30 °C causes whitening of brown adipose tissue in mice and decreases the expression of thermogenic genes [21]. Normal mouse body temperature is 37 °C, whereas normal goat body temperature is 39 °C [22]. In addition, a housing condition of 25 °C could significantly reduce the impact of cold stress on goat kids [23]. Therefore, we set 25 °C as thermoneutral condition of control group. In this study, compared with the room temperature group (25 °C), cold exposure (4 °C) significantly increased the protein levels of UCP1 and PGC1α. The individual cell compartment of perirenal BAT was reduced after cold exposure. These results suggest that room temperature (25 °C) can be considered a thermoneutral condition for the goats. Previous studies demonstrated that sympathetic nerves secrete catecholamines to stimulate β adrenergic receptors when mice are upon cold exposure, which will initiate the cAMP-PKA signaling pathway [24]. Then, phosphorylated PKA elicits the p38/MAPK signaling pathway and regulates the expression of BAT thermogenic genes [25]. In the present study, the cGMP-PKG but not cAMP signaling pathway was significantly enriched by KEGG analysis, suggesting that cold exposure may regulate goat BAT thermogenesis via the cGMP signaling pathway. The cGMP signaling pathway is characterized by activation of brown adipocytes differentiation and mitogenesis via p38/MAPK signaling pathway [26, 27]. Activation of the cGMP signaling pathway increases lipid uptake in BAT and increases the expression level of the *LPL* gene [28]. HSL directly catalyzes lipolysis after it is phosphorylated, and PLIN1 protects lipids from lipolysis but when phosphorylated uncouples protection from it. In addition, cGMP increases the phosphorylation levels of HSL and PLIN1, which then mediate lipolysis in adipocytes [29].

Fatty acid degradation can provide generous energy for BAT adaptive thermogenesis. In this study, we observed upregulated expression of *CPT1A*, *CPT2* as well as TCA cycle-related genes (*IDH2*, *OGDH,* and *DLST*) after cold exposure. FABP3, a key protein for lipid transport, is induced in BAT after cold exposure [30]. FABP3 also increases the uptake of free fatty acids from BAT in UCP1 knockout mice and promotes adaptive thermogenesis in BAT [31]. In addition, *CPT1A* and *CPT2* genes, which function to transport fatty acids into the inner mitochondrial membrane, are also significantly upregulated after cold exposure [32, 33]. Previous studies have reported that lipolytic products increase the expression of fatty acid oxidation-related genes through the PPAR signaling pathway [10]. Pyruvate dehydrogenase (PDH) can catalyze the reaction of catabolized pyruvate into acetyl CoA, thus promoting pyruvate into the TCA cycle [34]. Furthermore, previous studies proved that the insulin signaling pathway could activate the PDH of BAT [35]. Intermediates of the TCA cycle are significantly increased upon acute cold exposure, suggesting that the TCA cycle plays a critical role in energy metabolism for BAT thermogenesis [36]. Long-chain fatty acids, through β oxidation generated acetyl CoA, can enter the TCA cycle for oxidative degradation [37]. Our results demonstrate that fatty acid degradation and the TCA cycle pathways were activated after cold exposure, suggesting that perirenal BAT may provide energy for thermogenesis through these two metabolic pathways.

Cold exposure has previously been reported to remodel the lipid composition of interscapular BAT and increase the contents of very long-chain fatty acyls in SFA and MUFA [13]. Cold exposure increases the expression of the *ELOVL3* gene in interscapular BAT of mice [13, 20]. Our previous study also revealed that *ELOVL3* and *ELOVL5* genes are enriched in perirenal BAT compared to WAT [7]. However, there is no significant change for *ELOVL3* and *ELOVL5* genes after cold exposure in the present study. *ElOVL6* plays a regulatory role in the elongation of the chain length of fatty acids in interscapular BAT [38]. In this study, levels of fatty acetyl chains C20:0, C22:0, and C22:1 were increased in perirenal BAT after cold exposure. In addition, *ELOVL6* was significantly upregulated after cold exposure in perirenal BAT, indicating that *ELOVL6* may be necessary to prolong the fatty acid chain length under cold exposure.

Cold exposure can cause significant changes in mitochondrial phospholipid acyl chains composition in interscapular BAT via stimulation of β adrenergic receptors by sympathetic nerves [39]. The importance of cardiolipin for mitogenesis and BAT thermogenesis has been reported [14]. In this study, the expression of genes (*LPGAT1* and *CRSL1*) for cardiolipin synthesis were robustly activated. Cardiolipin was not detected in this study, and we speculate that there was a low content of cardiolipin in the brown fat of newborn goats. However, cardiolipin is enriched in interscapular BAT of adult mice [14]. Cardiolipin may be enriched in fat depots in adult goats, or this is a difference between goat and mouse brown fat. Moreover, the metabolism of glycerophospholipids is induced upon cold exposure in interscapular BAT and inguinal WAT [13, 40]. Mitochondria have a double membrane structure, in which PC and PE are the major components of membrane phospholipids [41]. Phospholipids on the mitochondrial membrane play an important role in maintaining mitochondrial metabolic function as well as UCP1 uncoupled respiration [42, 43]. Meanwhile, the increased amounts of PC and PE in mitochondria regulate the activity of mitochondrial enzymes and promote thermogenesis in BAT [43]. In the present study, we found that expression of genes of PC and PE synthesis were upregulated, and levels of PC and PE were increased in perirenal BAT after cold exposure. These results demonstrate that the level of PC and PE are increased upon cold exposure, which suggests their important role in adaptive thermogenesis in perirenal BAT. Finally, there were some limitations to our research. Metabolites and hormones in plasma are important for BAT thermogenesis [44, 45]. Although we identified some differentially expressed genes and lipid metabolites in BAT after cold exposure, the change of metabolites and hormones in plasma still need to be investigated. We will focus on plasma metabolites and hormones after cold exposure in the future research.

## Conclusion

We determined the changes of genes and lipid composition in goat BAT after acute cold exposure by RNA-seq and lipidomic analysis. These results revealed that cold exposure increased expression of genes involved in cGMP-PKG, TCA cycle, fatty acid elongation, and degradation pathways. We also found that cold exposure changes in lipid composition with a decrease in TG levels and an increase in PC and PE levels. These results indicate that the glycerolipids and glycerophospholipids pathways were activated in goat BAT after cold exposure. The data provides a reference to BAT thermogenesis regulation in large animals after cold exposure.

## Methods

### Animal and sampling

All animals were raised at the breeding center of Sichuan Agricultural University, Ya'an, China. A total of 10 female Chuanzhong black goats were artificially inseminated with the semen of a ram. The ewes delivered a total of 17 goat kids, including 11 males and 6 females. A total of 10 males were selected and randomized into room temperature (*n* = 5) and cold exposure groups (*n* = 5). After birth, the newborn goats were wiped and fed colostrum (30 mL/kg BW) in a 25 °C environment for 2 h. Then, kids from the room temperature group (RT) were kept at 25 °C and cold exposure (Cold) groups were placed into a cold room (6 °C) for 24 h. Warmed colostrum was fed three times at 8, 14, and 20 h of ages. After 24 h, all goats were sacrificed by arterial bleeding under full anesthesia. Perirenal adipose tissues were sampled and subsequently stored at -80 °C.

### Histology analysis

Perirenal fat were fixed with 4% paraformaldehyde and embedded in paraffin. For HE staining, sections were stained with hematoxylin (Solarbio, Beijing, China). Then, sections were photographed by the BX-50F light microscope (Olympus, Tokyo, Japan).

### Western blotting

Tissue lysate was obtained by the total protein extraction kit (Solarbio, Beijing, China). After protein samples transferring to PVDF membranes, we cut off the extra PVDF membrane according to the molecular weight of the target protein. Then, the remaining PVDF membrane was incubated with a primary antibody. Antibodies were diluted 1:500 for rabbit anti-UCP1 (Cat: 72,298; Cell Signaling Technology, MA, USA), 1:500 for rabbit anti-PGC1α (Cat: A12348; ABclonal, Wuhan, China), 1:1000 for rabbit anti-β-Tubulin (Cat: AC008; Abclonal, Wuhan, China), and 1:1000 for HPR-labeled goat anti-rabbit IgG (Beyotime, Shanghai, China). Finally, a ChemiDoc Imaging Systems (Bio-Rad, CA, USA) was used to detect immunoreactive proteins.

### RNA extraction and cDNA library construction

Total RNA was purified from adipose tissue with RNAisoPlus reagent (Takara, Tokyo, Japan). RNA integrity was assessed by the Agilent Bioanalyzer 2100 system (Agilent, CA, USA). Then, a total amount of 1 μg RNA per sample was enriched with magnetic beads with oligo (DT), after which the first cDNA strand was synthesized with random hexamers. The purified double-stranded cDNA was ligated for sequencing adaptors and finally sequenced by the novaseq 6000 (Illumina, CA, USA).

### RNA-seq analysis

FastQC was used to assess the quality of the sequencing data, after which clean reads were obtained [46]. The clean reads were mapped onto the goat reference genome by HISAT2 [47]. Reads on the alignment were assembled and quantified using StringTie [48]. Additionally, differential genes expression analysis was performed using edgeR and the significance of differential genes was set at *P* < 0.05 [49]. Finally, the GO and KEGG database were used for gene functional annotation and pathway enrichment analysis [16, 50]. KEGG pathway glycerophospholipid metabolism (ko00564), cGMP-PKG signaling pathway (ko04022), and cAMP signaling pathway (ko04024) were set as gene sets for gene set enrichment analysis (GSEA) [51].

### Lipidomics analyses

LC–MS analysis of perirenal fat was performed by the Agilent 1290 Infinity II liquid chromatography (UHPLC) system (Agilent, CA, USA) coupled with the triplet of 6600 mass spectrometer (AB SCIEX, Ma, USA). In brief, samples were extracted with chloroform–methanol mixed solution (70:30, v/v). Chromatographic separation was used with a Phenomenex Kinetex C18 column (Phenomenex, CA, USA). Eluent A was consisted of acetonitrile and water (40:60, v/v). Eluent B was consisted of isopropanol and acetonitrile (90:10, v/v), and then the procedure was carried as previous study [52]. The mass spectrometry parameters were used as follows: ion source gas 1 (60 psi), ion source gas 2 (60 psi), curtain gas (30 psi), and temperature of 600 °C; ion spray voltage floating (ISVF), 5000 V or -4500 V in positive or negative modes, respectively.

Using R package Lipidview (https://github.com/luechtian/LipidView_analysis) for lipid identification and annotation, a data matrix of peak response values (intensity) was obtained. The data were normalized by raw peak area values/total peak area values. The acyl chain content was calculated by summing the content of that acyl chain in all individual TG species. The orthogonal partial least squares-discriminant analysis (OPLS-DA) was used to analyzed lipids data by R package ropls (version 1.3.16). Differences in the lipid between the control group and the L-carnitine treatment group were determined using the Mann–Whitney U test. Lipids with *P* < 0.05 were set as significantly changed.

## Supplementary Information

**Additional file 1:** **Table S1.** Information of RNA-seq

**Additional file 2:** **Table S2.** Information of differentially expressed genes between RT and Cold group.

**Additional file 3:** **Table S3.** Information of enriched KEGG terms of  upregulated genes by cold exposure.

**Additional file 4:** **Table S4.** Values for all the lipid class

**Additional file 5:** **Table S5.** Values for the significantly different lipid class

**Additional file 6:** **Table S6.** The length of fatty acyl chains associated with TG

**Additional file 7:**  **Fig. S1.** Whole membrane images for Fig. 1B

## Data Availability

All data generated in this study are included in the main article and its supplementary files. All the raw sequencing data have been deposited in the NCBI Sequence Read Archive (SRA) database (https://dataview.ncbi.nlm.nih.gov/object/PRJNA821931?reviewer=d830vdm7of273lbk6m6ko70rr1).

## Abbreviations

BAT: Brown adipose tissue
WAT: White adipose tissue
FPKM: Fragments Per Kilobase Million
FDR: False discovery rate
GO: Gene Ontology
KEGG: Kyoto Encyclopedia of Genes and Genomes
OPLS-DA: Orthogonal partial least squares-discriminant analysis
TG: Triglyceride
DG: Diglyceride
MG: Monoglyceride
CE: Cholesterol ester
LPA: Lysophosphatidic acid
PA: Phosphatidic acid
LPC: Lysophosphatidylcholine
PC: Phosphatidylcholine
LPE: Lysophosphatidylethanolamine
PE: Phosphatidylethanolamine
LPG: Lysophosphatidylglycerol
PG: Phosphatidylglycerol
LPI: Lysophosphatidylinositol
PI: Phosphatidylinositol
LPS: Lysophosphatidylserine
PS: Phosphatidylserine
SM: Sphingomyelin
Cer: Ceramide
CerP: Ceramides phosphate
Phytocer: Phytoceramide
LacCer: Lactosyl ceramide
SPH: Sphingosine
GlcCer: Glucosylceramide
