# Supplementary material for: Cold exposure induces lipid dynamics and thermogenesis in brown adipose tissue of goats
Source: BMC Genomics. 2022 Jul 21;23:528. doi: 10.1186/s12864-022-08765-5 (PMC9306100; doi:10.1186/s12864-022-08765-5)

Exposure 5.0 sec

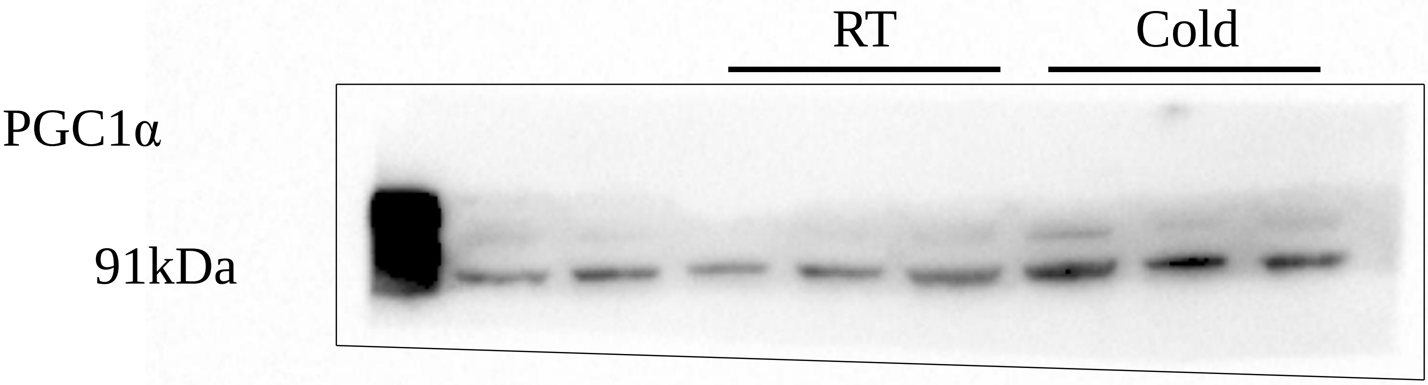

Exposure 7.0 sec

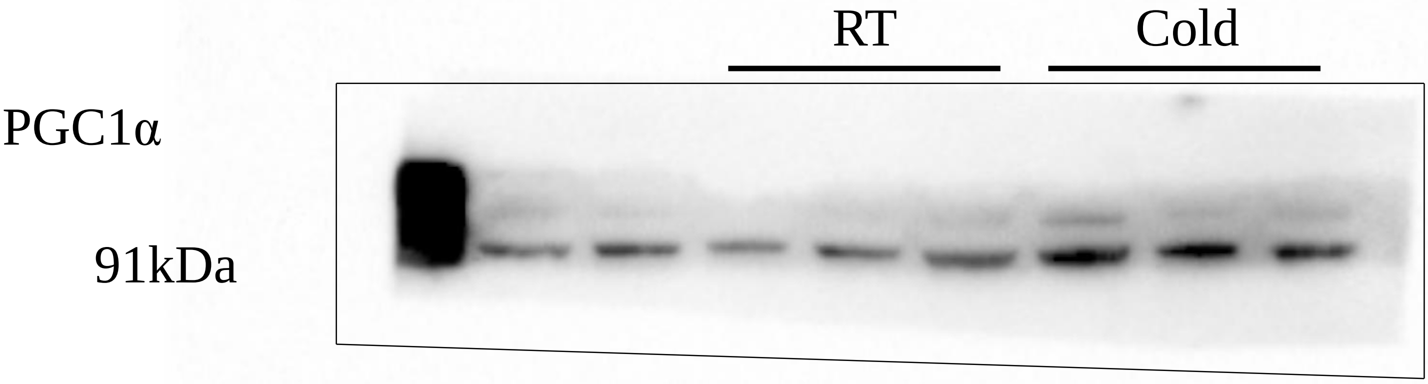

Exposure 11.0 sec

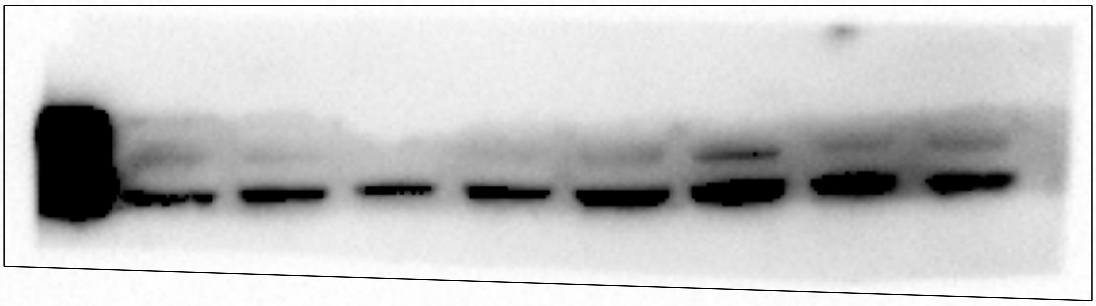

Exposure 14.0 sec

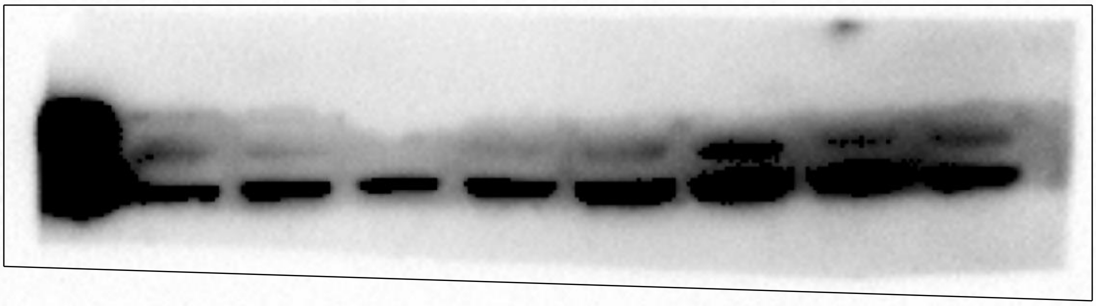

Exposure 10.0 sec

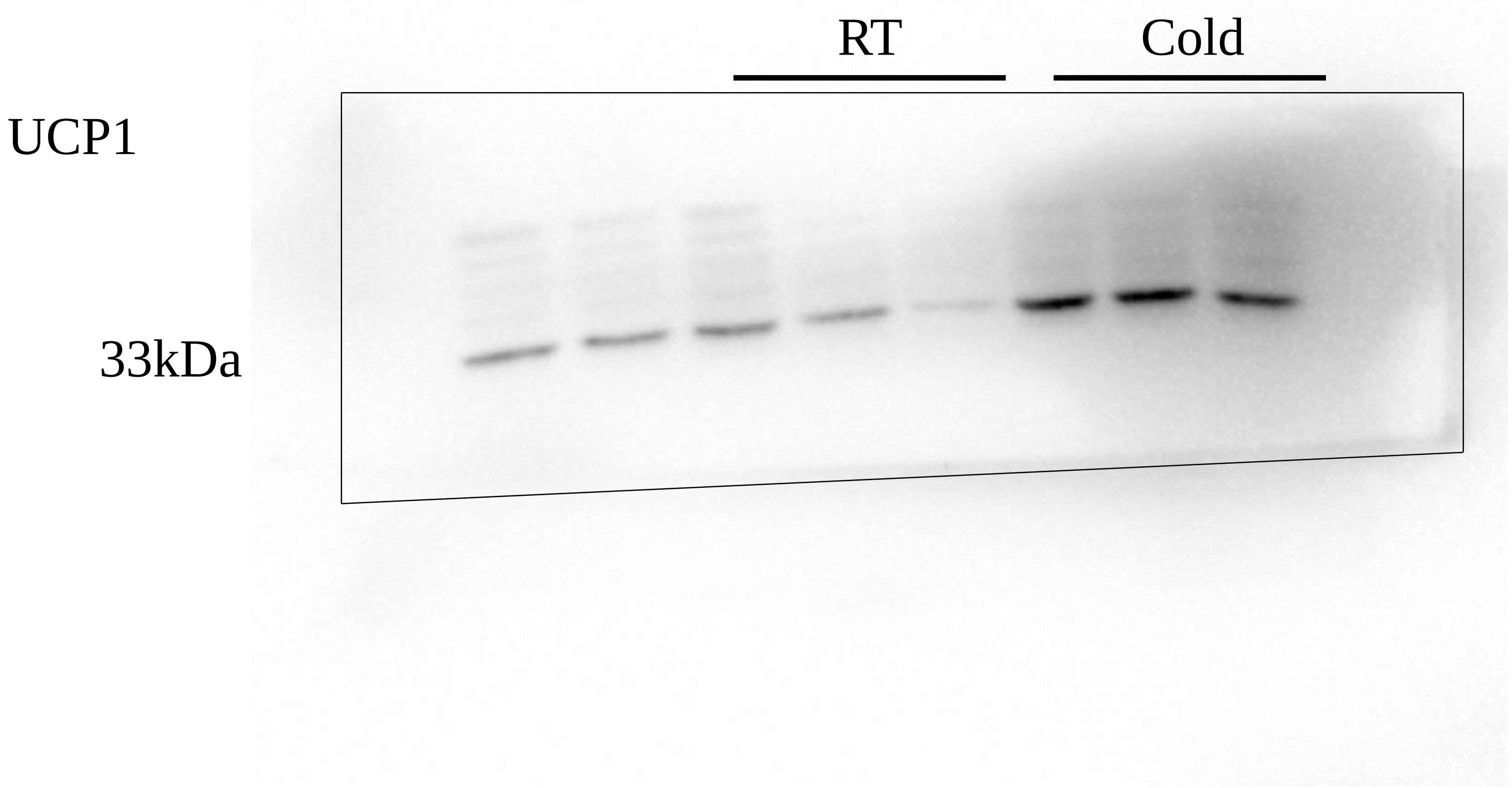

Exposure 13.0 sec

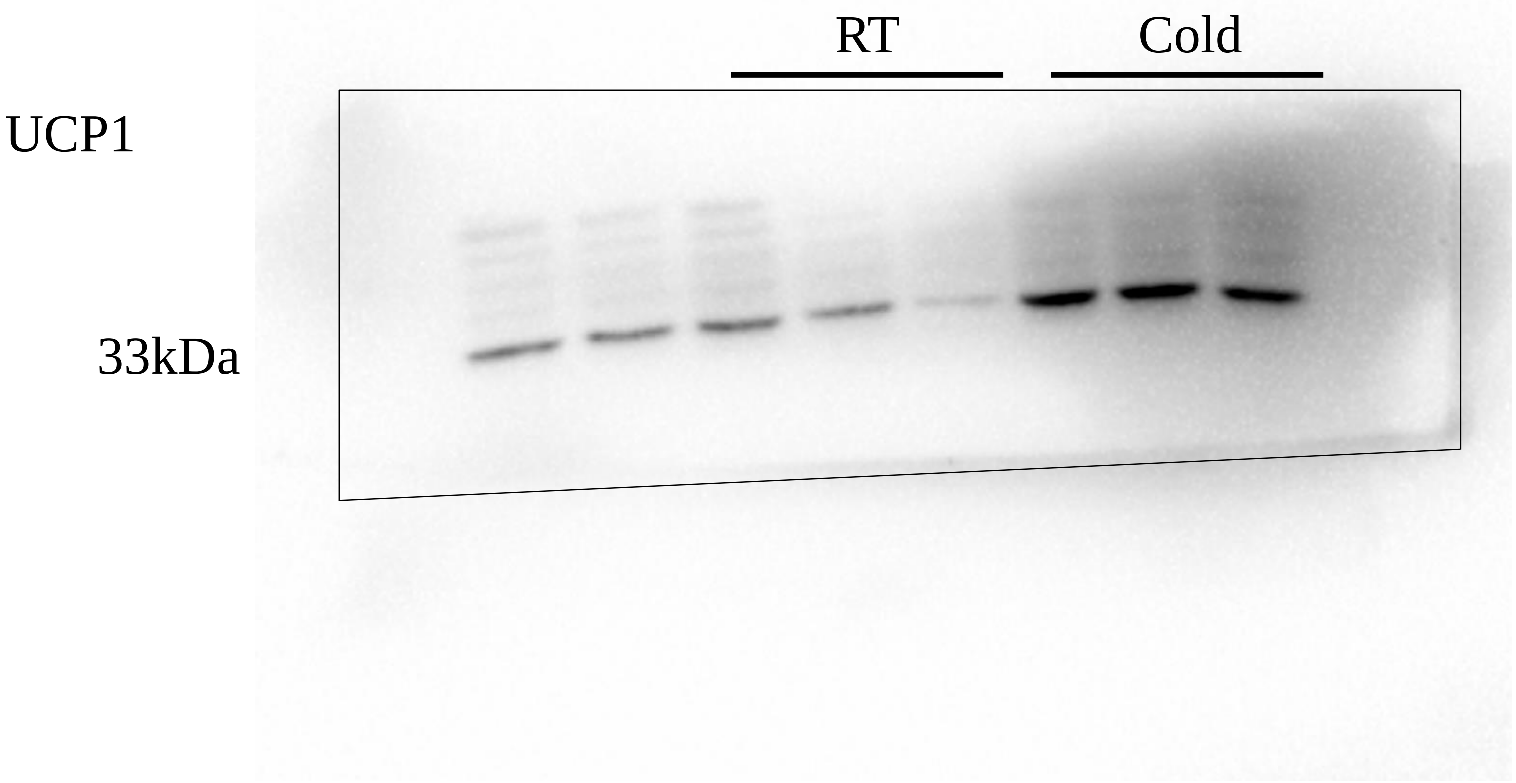

Exposure 17.0 sec

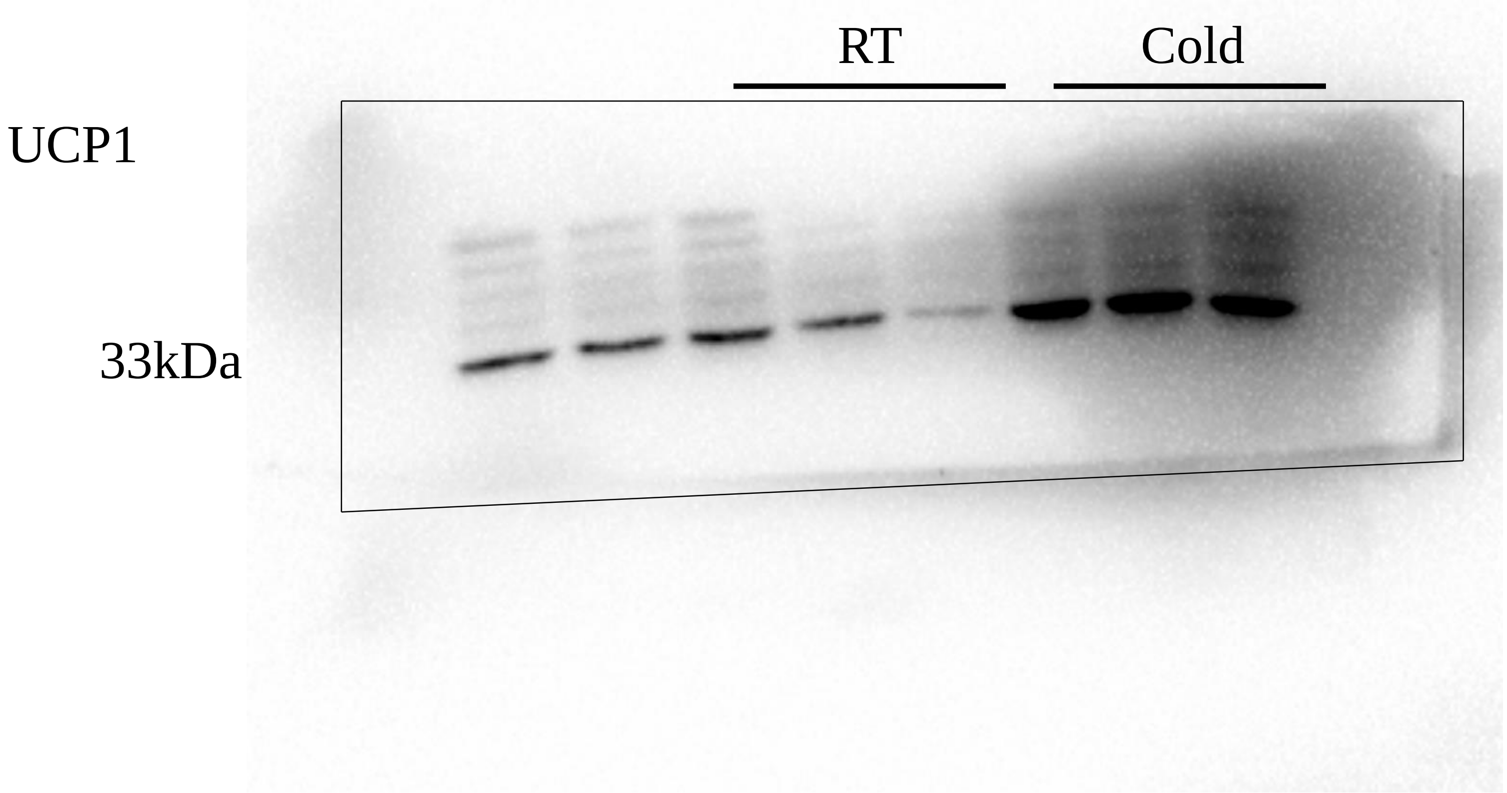

Exposure 19.0 sec

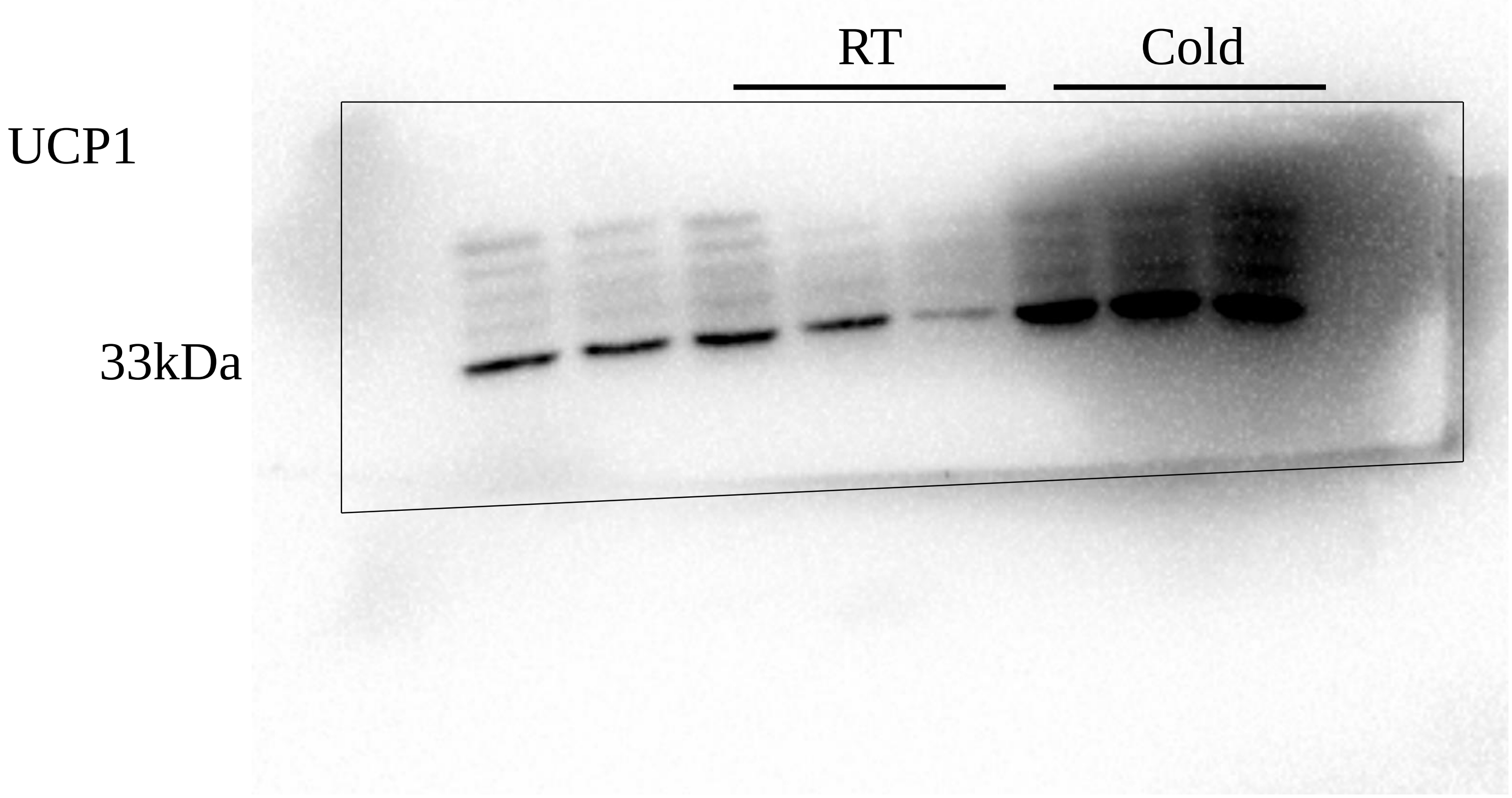

Exposure 11.0 sec

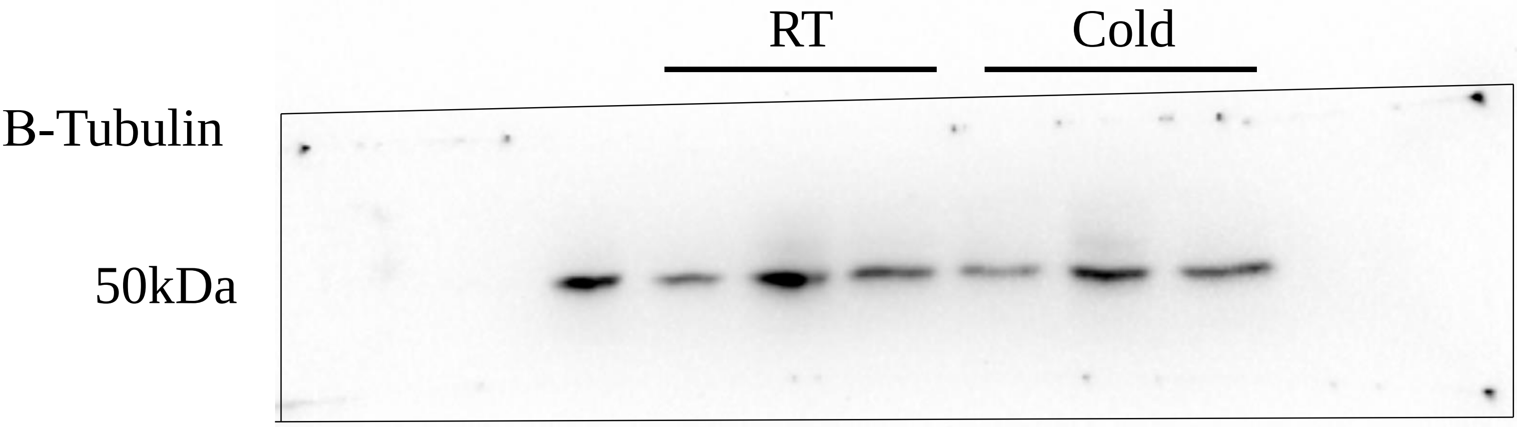

Exposure 14.0 sec

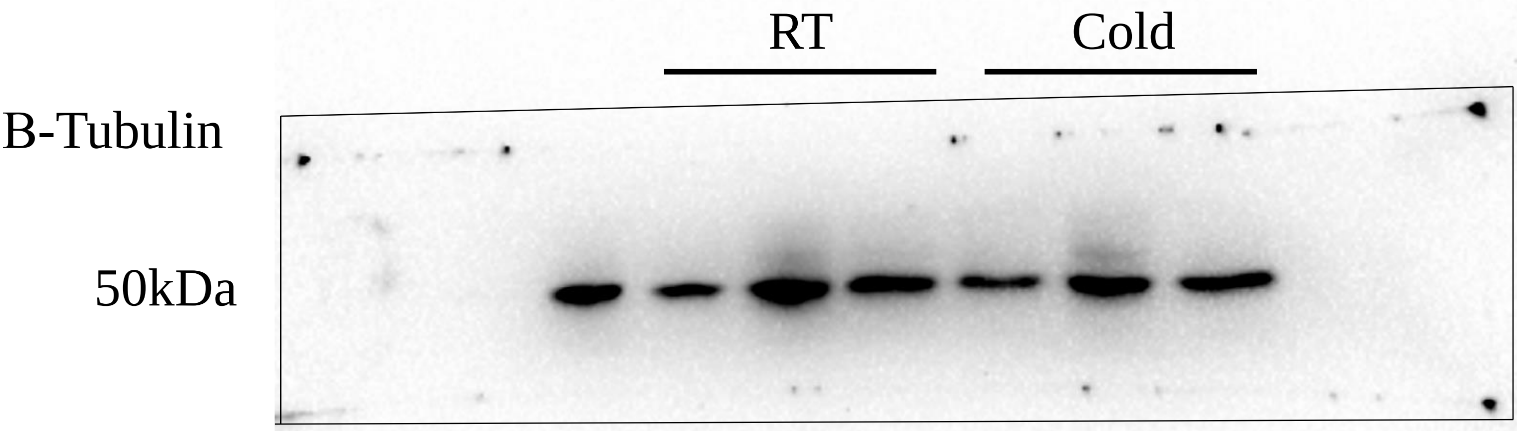

Exposure 18.0 sec

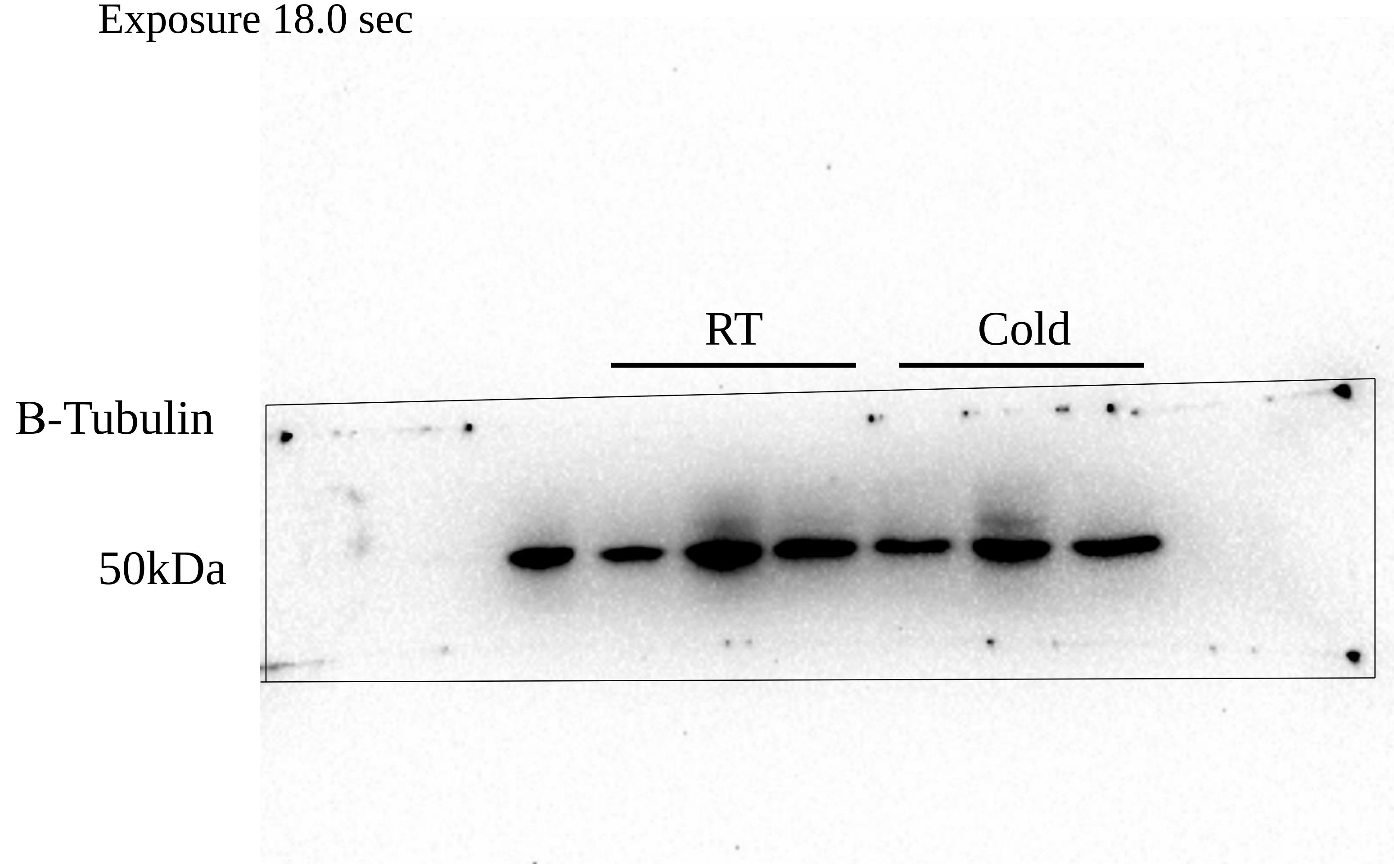

Exposure 21.0 sec

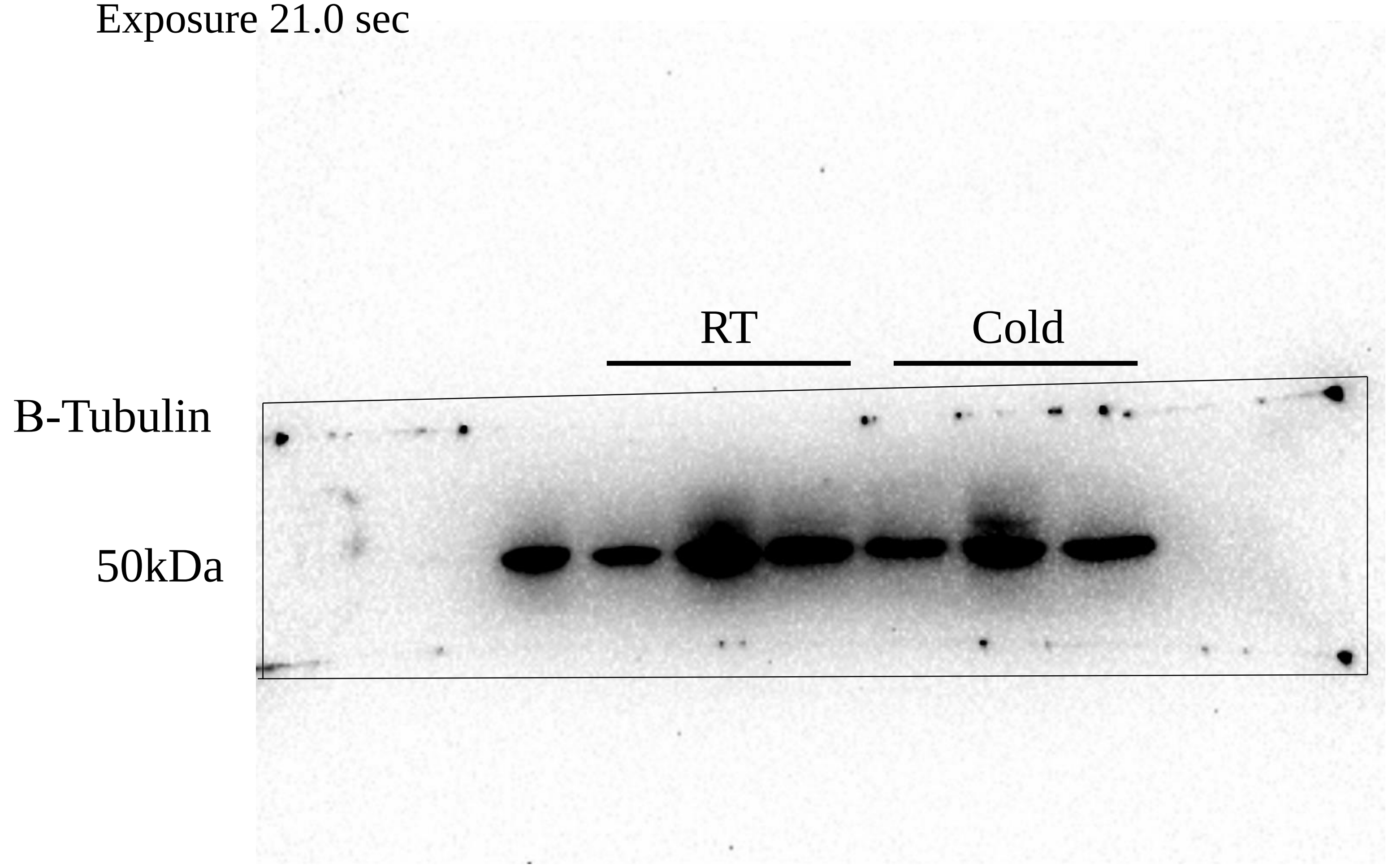

Supplement: Supplementary file 7 — Additional file 7: Fig. S1. Whole membrane images for Fig. 1B [file 12864_2022_8765_MOESM7_ESM.pdf]
